# Bis(Triphenylamine)Benzodifuran Chromophores: Synthesis, Electronic Properties and Application in Organic Light-Emitting Diodes

**DOI:** 10.3389/fchem.2021.721272

**Published:** 2021-07-21

**Authors:** Hui Li, Ryutaro Komatsu, Jihane Hankache, Hisahiro Sasabe, Latevi Max Lawson Daku, Bilal Özen, Songjie Chen, Jürg Hauser, Andreas Hauser, Silvio Decurtins, Junji Kido, Shi-Xia Liu

**Affiliations:** ^1^ Department of Chemistry, Biochemistry and Pharmaceutical Sciences, University of Bern, Bern, Switzerland; ^2^ Department of Organic Device Engineering Research Center for Organic Electronics, Yamagata University, Yamagata, Japan; ^3^ Department of Physical Chemistry, University of Geneva, Geneva, Switzerland

**Keywords:** benzodifuran, triphenylamine, spectroelectrochemistry, optical spectroscopy, organic light-emitting diode

## Abstract

A series of bis(triphenylamine)benzodifuran chromophores have been synthesized and fully characterised. Starting from suitably functionalized benzodifuran (BDF) precursors, two triphenylamine (TPA) moieties are symmetrically coupled to a central BDF unit either at 4,8-positions through double bonds (**1**) and single bonds (**2**) respectively, or at 2,6-positions through double bonds (**3**). Their electronic absorption and photoluminescence properties as well as redox behaviour have been investigated in detail, indicating that the π-extended conjugation *via* vinyl linkers in **1** and **3** leads to comparatively strong electronic interactions between the relevant redox moieties TPA and BDF. Due to intriguing electronic properties and structural planarity, **3a** has been applied as a dopant emitter in organic light-emitting diodes. A yellowish-green OLED exhibits a high external quantum efficiency (EQE) of 6.2%, thus exceeding the theoretical upper limit most likely due to energy transfer from an interface exciplex to an emissive layer and/or favorable horizontal orientation.

## Introduction

**SCHEME 1:**
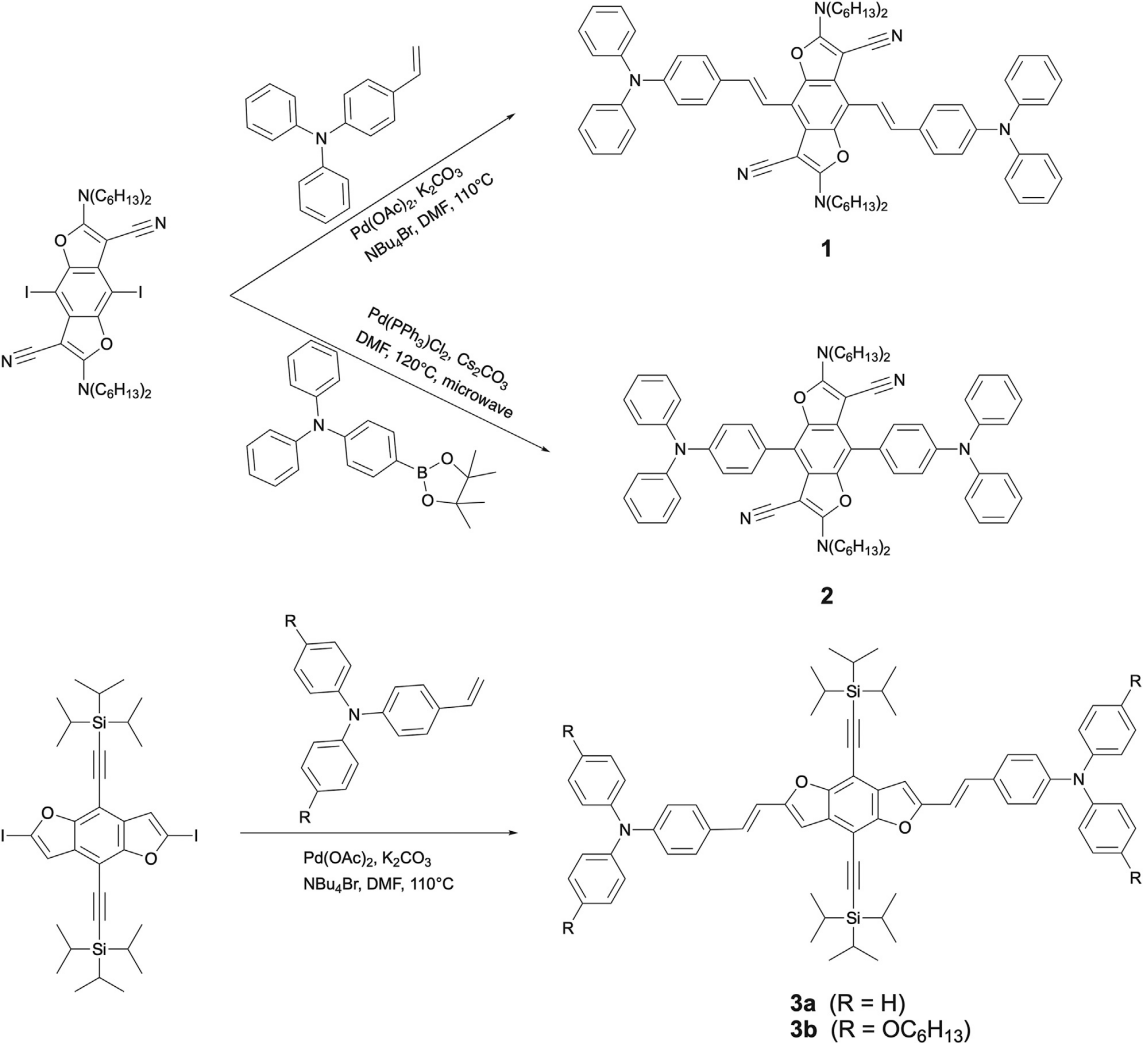
Synthetic routes to triads **1**, **2,** and **3**.

Benzodifuran (BDF) derivatives are of particular interest and relevance for applications as organic semiconductors due to their structural and electronic features. The symmetry and planarity as well as π-extended conjugation can enhance electron delocalization and intermolecular interactions and thus improve charge mobility. After recent explorations of facile and efficient synthetic methodologies, BDF derivatives, including small molecules and polymers, have been widely used for high performance organic electronics such as organic photovoltaics (Li et al., 2010; Huo et al., 2012a; Huo et al., 2012b; Li et al., 2012; Aeschi et al., 2013; Li et al., 2013b; Kularatne et al., 2013; Gao et al., 2020), organic field effect transistors (OFETs) (Huang et al., 2014; Wang et al., 2016; Zhang et al., 2017; Wang et al., 2018), organic light-emitting diodes (OLEDs) (Tsuji et al., 2007; Tsuji et al., 2009; Mitsui et al., 2012) and single-molecule devices (Li et al., 2014; Huang et al., 2015; Xiang et al., 2015; Baghernejad et al., 2020). It has been demonstrated that extended π-conjugated BDF derivatives suitably functionalised with either pyridine (Yi et al., 2010), or pyrene and anthracene (Keller et al., 2011) or triphenyl amine (TPA) (Faurie et al., 2018) termini show very strong fluorescence emission with luminescence quantum yields Φ_F_ of up to 0.98. Inspired by these results we have explored new chromophores for high performance OLED applications, utilising the synergetic effect of the BDF core and two TPA substituents linked either through single bonds or double bonds along the long and the short axes (Scheme 1).

**FIGURE 1:**
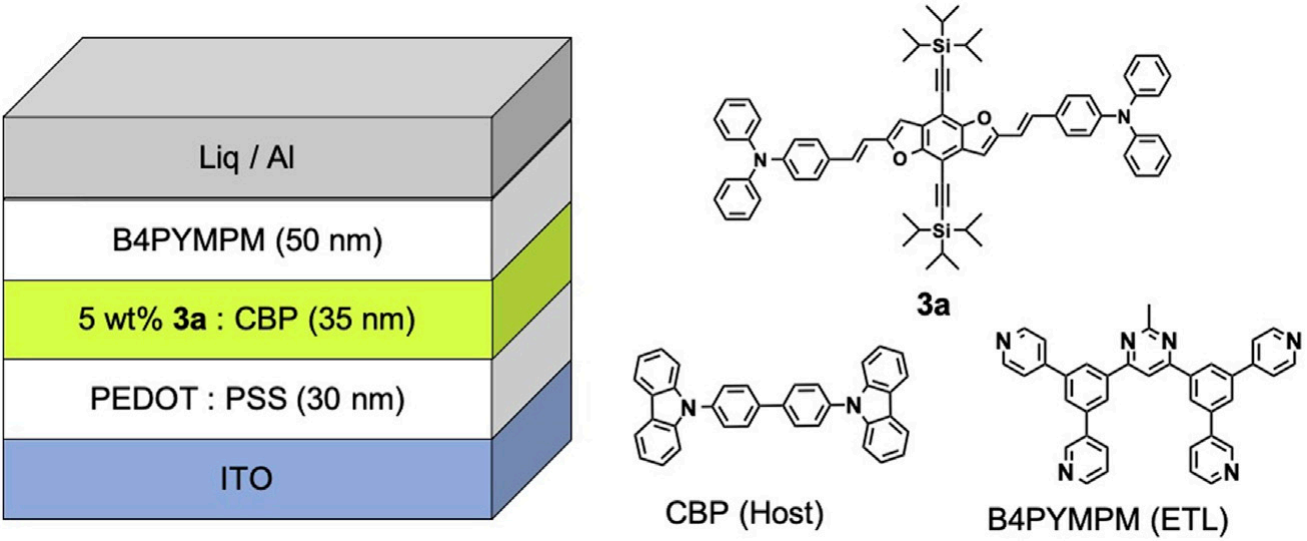
The configuration of the OLED device and the chemical structures of **3a**, CBP and B4PyMPM.

OLEDs have attracted significant attention because of their promising applications in flexible large-scale flat-panel displays and their potential for low cost fabrication (Baldo et al., 2000; Wu et al., 2004; Burn et al., 2007; de Sousa et al., 2020; Xu et al., 2021). The characteristic organic semiconducting materials in OLED devices will realise good flexibility over a large-area fabrication and high-performance optical and electrical properties. As one of the most important parameters for the device, the external quantum efficiency (EQE) of fluorescent OLEDs is determined by several factors, including the charge balance of the holes and the electrons, the singlet exciton ratio, the photoluminescence quantum efficiency (PLQE) and the light-outcoupling efficiency (Thejo Kalyani and Dhoble, 2012; Poriel and Rault-Berthelot, 2020). With the limit of the singlet exciton ratio of 25% and the upper theoretical limit of the EQE of 5%, the great challenge has indeed been to improve the EQE of fluorescent OLED devices. In recent studies, the charge balance of holes and electrons has been investigated for elucidating how to affect and maximise the EQE (Pu et al., 2012; Wei et al., 2018; Teng et al., 2020). Although the application of exciplex formation for tuning emission colors (Cheng et al., 2005) or for obtaining white OLEDs (Kalinowski et al., 2007; Tong et al., 2007) has been pursued, there are only few reports concerning the enhancement of the EQE by energy transfer from exciplexes in OLED devices (Park et al., 2011; Park et al., 2013; Kim & Kim, 2019). In this paper, we describe the synthesis, redox and photophysical properties of BDF-TPA triads, **1–3** (Scheme 1). Among them, **3a** holds the promise of a better OLED performance in term of intriguing electronic properties and structural planarity. Therefore, only **3a**-based OLED devices have been investigated in detail. The yellow-greenish OLED device using 1 wt% **3a**-doped 4,4′-bis(N-carbazolyl)-1,1′-biphenyl (CBP, Figure 1) as an emissive layer shows a low turn-on voltage of 3.2 V and an EQE of 6.2%, which is 35% higher than the theoretical EQE upper limit. This is very probably due to energy transfer from an exciplex formed between an electron transporting layer and the host materials to the emissive layer.

## Experimental

### Materials and Methods

2,6-Bis(*N*,*N*-dihexylamino)-4,8-diiodobenzo[1,2-*b*:4,5-*b*′] difuran-3,7-dicarbonitrile (Yi et al., 2010) and 4,8-bis(triisopropylsilylethynyl)-2,6-diiodobenzo[1,2-*b*:4,5-*b*′]difuran (Huang et al., 2015) were previously reported. 4-(4,4,5,5-Tetramethyl-[1,3,2]dioxaborolane)phenyl -*N*,*N*-diphenylamine (Fan et al., 2012), *N*-phenyl-*N*-(4-vinylphenyl)aniline (Zhou et al., 2011) and 4-(hexyloxy)-*N*-[4-(hexyloxy)phenyl]-*N*-(4-vinylphenyl)aniline (Ko et al., 2012) were synthesized according to the literature procedures. All the chemicals and solvents were purchased from commercial sources and were used without further purification, unless specially mentioned. All the reactions were carried out, unless mentioned, under normal laboratory conditions in air. Microwave reactions were conducted using the Biotage Initiator Eight EXP microwave apparatus and the corresponding vials. The reaction was performed in a glass vial (capacity 20 ml) sealed with a septum, under magnetic stirring. The temperature of the reaction mixture was monitored using a calibrated infrared temperature control mounted under the reaction vial. ^1^H NMR spectra were obtained on a Bruker AC 300 spectrometer operating at 300 MHz. Chemical shift in ppm is quoted relative to residual solvent signals calibrated as follows: δ(CDCl_3_) = 7.26 ppm, δ(CD_2_Cl_2_) = 5.32 ppm. Multiplicities in the ^1^H NMR spectra are described as: s = singlet, d = doublet, t = triplet, m = multiplet; coupling constants are reported in Hz. Mass spectra were recorded either with ESI (electrospray ionization) on a Thermo Scientific LTQ Orbitrap XL in the positive mode or with Matrix assisted Laser desorption ionization, coupled to a Time-of-Flight analyzer (MALDI-TOF) on a Ultraflex spectrometer.

### Synthetic Procedures

#### Synthesis of 1

A mixture of 2,6-bis(*N*,*N*-dihexylamino)-4,8-diiodobenzo[1,2-*b*:4,5-*b*′]difuran-3,7-dicarbonitrile (317.2 mg, 0.384 mmol), *N*-phenyl-*N*-(4-vinylphenyl)aniline (260 mg, 0.96 mmol), K_2_CO_3_ (132.6 mg, 0.96 mmol), NBu_4_Br (123.7 mg, 0.384 mmol), Pd(OAc)_2_ (17 mg, 0.076 mmol) and anhydrous DMF (20 ml) was bubbled with N_2_ for 10 min and then the resulting solution was heated at 110°C overnight in an inert atmosphere. The volatile was evaporated by rotavapor and the residue was subjected to column chromatography on silica gel, eluting with a gradient of CH_2_Cl_2_ and hexane (1:1 to 1:0) to afford an orange crystalline product (273.7 mg, 64%). ^1^H NMR (300 MHz, CDCl_3_) δ 7.89 (d, *J* = 16.4 Hz, 2H), 7.43 (m, 6H), 7.26 (m, 8H), 7.12 (m, 8H), 7.03 (m, 8H), 3.63 (m, 8H), 1.80 (m, 8H), 1.36 (m, 24H), 0.87 (t, *J* = 6.9 Hz, 12H). ESI-MS: Calc. for C_76_H_84_N_6_O_2_ [M]^+^ 1112.67, found: 1112.66; [M]^2 +^ 556.34, found: 556.33.

#### Synthesis of 2

A mixture of 4-(4,4,5,5-tetramethyl-[1,3,2]dioxaborolane) phenyl-*N*,*N*-diphenylamine (317 mg, 1 mmol), 2,6-bis(*N*,*N*-dihexylamino)-4,8-diiodobenzo[1,2-*b*:4,5-*b*′]difuran-3,7-di- carbonitrile (330 mg, 0.4 mmol), Pd(PPh_3_)_2_Cl_2_ (140 mg, 0.2 mmol) and Cs_2_CO_3_ (326 mg, 1 mmol) in DMF (6 ml)/H_2_O (1 ml) was added to a 20 ml microwave vial. The reactor was sealed and purged with Ar for 20 min. The resulting mixture was subjected to microwave irradiation by prestirring for 1 min and reacted at 120°C for 60 min. The resulting mixture was poured into water (10 ml) and extracted with CH_2_Cl_2_ (20 ml). The volatile was evaporated by rotavapor, and the residue was subjected to column chromatography on silica gel, eluting with 1:1 CH_2_Cl_2_/hexane, to afford a light-yellow solid product (301 mg, 71%). ^1^H NMR (300 MHz, CDCl_3_) δ: 7.45 (d, *J* = 8.7 Hz, 4H), 7.23 (m, 8 H), 7.17 (m, 12H), 7.04 (m, 4H), 3.47 (t, *J* = 7.74 Hz, 8H), 1.66 (m, 8H), 1.27 (m, 24H), 0.85 (m, 12H). ^13^C NMR (75.5 MHz, CDCl_3_) δ: 129.21, 124.70, 47.86, 31.59, 31.20, 26.56, 25.91, 22.68, and 14.03. MALDI-TOF MS: Calcd. for C_72_H_80_N_6_O_2_ [M]^+^ 1060.63, found: 1060.75.

#### Synthesis of 3a

A mixture of *N*-phenyl-*N*-(4-vinylphenyl)aniline (339 mg, 1.25 mmol), 4,8-bis(triisopropylsilylethynyl)-2,6-diiodobenzo[1,2-*b*:4,5-*b*′]difuran (385 mg, 0.5 mmol), anhydrous potassium carbonate (172 mg, 1.25 mmol), tetrabutylammonium bromide (161 mg, 0.5 mmol), and Pd(OAc)_2_ (22 mg, 0.01 mmol) was added into anhydrous DMF (30 ml) under N_2_ atmosphere. The resulting mixture was stirred at 110°C for 4 h, and then cooled to room temperature, followed by pouring into water (400 ml) with stirring. The mixture was extracted with CH_2_Cl_2_, and the organic phase was combined and washed with brine. After drying with anhydrous MgSO_4_, the solvent was removed. The crude product was purified by column chromatography (silica gel) using hexanes/dichloromethane (4:1) as eluent to obtain the product as an orange powder (250 mg). Yield 47%; ^1^H NMR (300 MHz, CD_2_Cl_2_) δ 7.42 (d, *J* = 8.7 Hz, 4H), 7.32 (m, 10H), 7.13 (m, 16H), 6.97 (d, *J* = 15.9 Hz, 2H), 6.82 (s, 2H), 1.25 (s, 42H); ^13^C NMR (75.5 MHz, CDCl_3_) δ 156.99, 152.48, 148.42, 147.53, 131.07, 130.34, 129.54, 127.92, 125.11, 123.66, 122.96, 113.98, 104.62, 100.96, 99.28, 98.28, 18.77, and 11.61; MALDI-TOF MS: Calcd. for C_72_H_76_N_2_O_2_Si_2_ [M]^+^ 1056.54, found: 1056.54. HRMS: Calcd. for C_72_H_76_N_2_O_2_Si_2_ [M]^+^ 1056.5445, found: 1056.5448.

#### Synthesis of 3b

Following the same synthetic procedure for **3a**, *N*-phenyl-*N*-(4-vinylphenyl)aniline was replaced by 4-(hexyloxy)-*N*-(4-(hexyloxy)phenyl)-*N*-(4-vinylphenyl)aniline (590 mg, 1.25 mmol). The reaction mixture was stirred under 110°C for 6 h. After cooling down to room temperature, the mixture was poured into water and extracted with CH_2_Cl_2_. The organic phase was evaporated and purified on silica gel by flash chromatography using a gradient of hexanes:CH_2_Cl_2_ (1:0 to 1.5:1). The pure product was isolated as a red powder (0.36 g). Yield 49%; ^1^H NMR (300 MHz, CDCl_3_) δ 7.32 (d, *J* = 8.6 Hz, 4H), 7.07 (d, *J* = 7.4 Hz, 8H), 6.85 (d, *J* = 8.8 Hz, 14H), 3.95 (t, *J* = 6.5 Hz, 8H), 1.79 (m, 8H), 1.37 (m, 24H), 1.26 (s, 42H) 0.92 (t, *J* = 7.0 Hz, 12H); ESI-MS: Calcd. for C_96_H_124_N_2_O_6_Si_2_ [M]^+^ 1456.90, found: 1456.90; [M]^2 +^ 728.45, found: 728.45.

### Electrochemistry

Cyclic voltammetry measurements in CH_2_Cl_2_ (10^−4^ M) were performed at room temperature under Ar with a three-electrode cell, using 0.1 M Bu_4_NPF_6_ as supporting electrolyte, an Ag/AgCl electrode containing 2 M LiCl (in ethanol) as reference electrode, a glassy carbon electrode as counter electrode, and a Pt disk as working electrode at a scan rate of 100 mV/s.

### Spectroscopic Measurements

High quality absorption spectra of all compounds in CH_2_Cl_2_ were recorded in 1 cm quartz cells with a Cary 5000 dual beam spectrometer at concentrations of 1 × 10^−5^ M. PL and excitation spectra in CH_2_Cl_2_ of the same solutions were recorded with a Fluorolog 3 spectrometer.

Spectroelectrochemical measurement were performed using an optically transparent thin layer cell (d = 0.7 mm) from Specac, which was placed inside the Cary 5000 spectrometer, and the electrochemical potential was applied by using a potentiostat in three electrode configuration. All potentials are given *versus* the silver wire pseudo-reference electrode.

### Computational Details

DFT (Hohenberg and Kohn, 1964; Kohn and Sham, 1965) and TD-DFT (Casida and Chong, 1995) based calculations on **1** were performed with the B3LYP functional (summer 1994; Becke, 1993) and the DZVP2 basis (Godbout et al., 1992) set using the NWChem program package (Aprà et al., 2020). Optimization calculations were performed using tight convergence criteria and the subsequent TDDFT electronic calculations were performed within the Tamm-Dancoff approximation (Hirata and Head-Gordon, 1999).

### Crystallography

A crystal of **2** was mounted with Apiezon on a glass needle and used for X-ray structure determination at −100°C. All measurements were made on an *Oxford Diffraction SuperNova* area-detector diffractometer (2010) using mirror optics monochromated Mo *K*α radiation (λ = 0.71073 Å). The unit cell constants and an orientation matrix for data collection were obtained from a least-squares refinement of the setting angles of 5,150 reflections in the range 2.10° < θ < 25.30°. A total of 1,300 frames were collected using *ω* scans, 120 s exposure time and a rotation angle of 0.5° per frame, and a crystal-detector distance of 50.0 mm.

Data reduction was performed using the (CrysAlisPro, 2010) program. The intensities were corrected for Lorentz and polarization effects, and an absorption correction based on the multi-scan method using SCALE3 ABSPACK in (CrysAlisPro, 2010) was applied. Data collection and refinement parameters are given in Supplementary Table 1.

The structure was solved by direct methods using *SUPERFLIP* (Palatinus and Chapuis, 2007), which revealed the positions of all non-hydrogen atoms of the title compound. The non-hydrogen atoms were refined anisotropically. All H-atoms but the ones from water were placed in geometrically calculated positions and refined using a riding model where each H-atom was assigned a fixed isotropic displacement parameter with a value equal to 1.2 Ueq of its parent atom (1.5 Ueq for the ammonium groups).

Refinement of the structure was carried out on *F* ^*2*^ using full-matrix least-squares procedures, which minimized the function Σw(*F*_o_^2^ – *F*_c_^2^)^2^. The weighting scheme was based on counting statistics and included a factor to downweight the intense reflections. All calculations were performed using the *SHELXL-97* (Sheldrick, 2008) program.

### Device Fabrication

A solution processed OLED was fabricated with a structure of [ITO (130 nm)/PEDOT:PSS (40 nm)/**3a** (1 and 5 wt%) doped CBP (30 nm)/B4PYMPM (50 nm)/Liq (3 nm)/Al (100 nm)]. A 40 nm of PEDOT:PSS (CH8000) film as a hole injection layer (HIL) was spin-coated on an ITO substrate and annealed at 200°C in air. Then, a 30 nm of 1 or 5 wt% **3a**-doped CBP film as an emitting layer (EML) was spin-coated from a THF solution (5 mg ml^−1^) and annealed at 60°C for 10 min B4PYMPM as an electron-transporting layer (ETL) and Liq/Al as a cathode were thermally evaporated under vacuum. The current density (J) – luminance (L) – voltage (V) characteristics of the OLEDs were measured by a Keithley source meter 2,400 and a Konica Minolta CS-200, respectively. Electroluminescence (EL) spectra were taken by an optical multichannel analyzer, Hamamatsu PMA 11. The angular dependence of luminous intensity was measured using a Keithley source measure unit 2,400 and a Minolta CS2000. External quantum efficiencies were calculated from the front luminance, current density and EL spectrum.

## Results and Discussion

### Synthesis and Characterization

**FIGURE 2:**
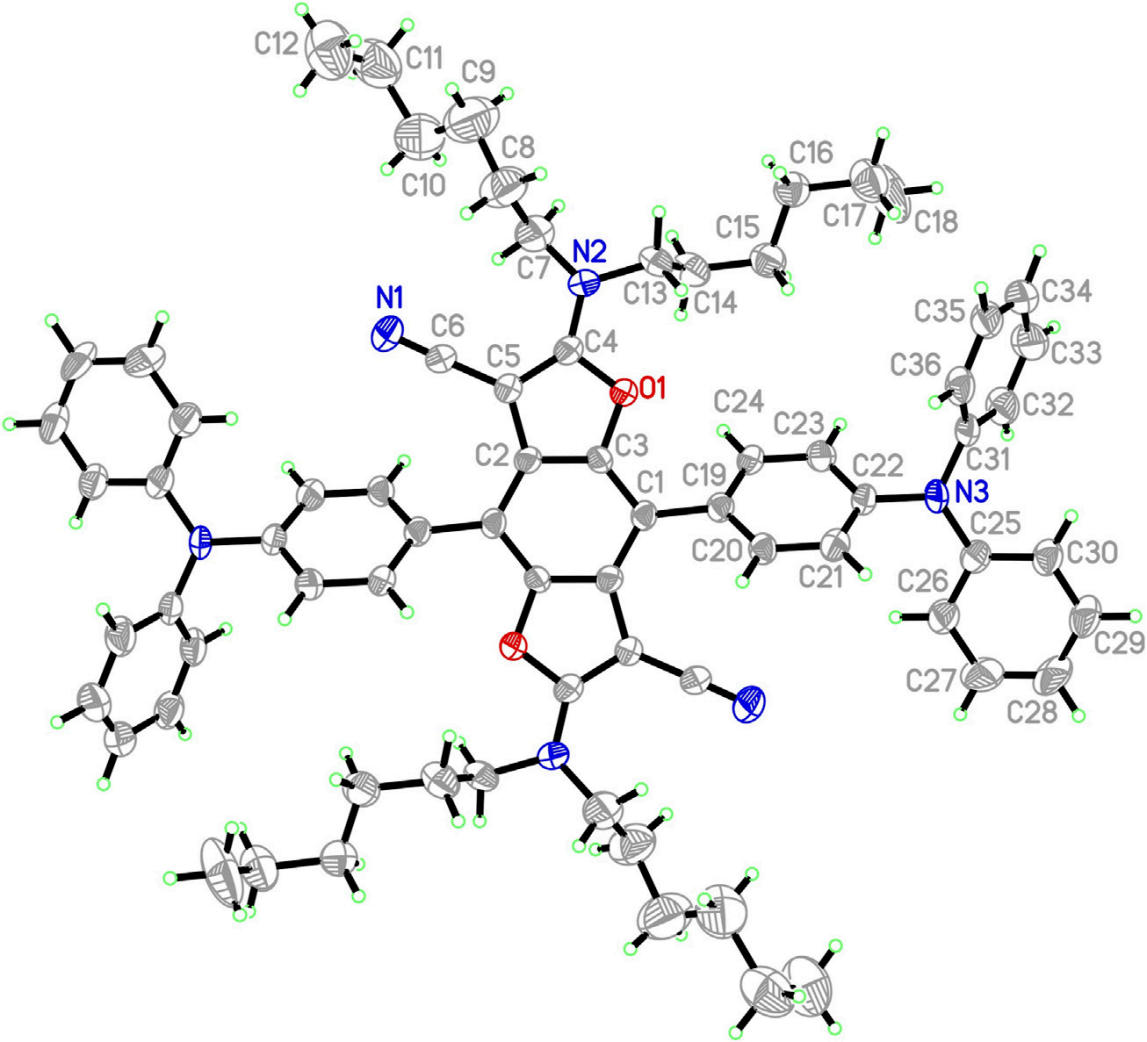
ORTEP representation of one independent molecule **2** (50% probability ellipsoids; H-atoms given arbitrary displacement parameters for clarity).

The synthetic routes to triads **1**, **2,** and **3**, in which two TPA moieties are symmetrically coupled to a central BDF unit, are outlined in Scheme 1. Starting from 2,6-bis(*N*,*N*-dihexylamino)-4,8-diiodobenzo[1,2-*b*:4,5-*b*′]difuran-3,7-dicarbonitrile (Yi et al., 2010), the target compounds **1** and **2** were prepared in good yields *via* palladium-catalyzed Heck and Suzuki cross-coupling reactions with the corresponding TPA precursors, respectively. Similarly, triads **3** were readily obtained from 4,8-bis(triisopropylsilylethynyl)-2,6-diiodobenzo[1,2-*b*:4,5-*b*′]difuran in reasonable yields by reactions with 2.5 equivalents of the corresponding TPA derivatives under standard Heck cross-coupling conditions. The structural planarity and π-extended conjugation between redox active TPA and BDF units through vinyl spacers are the same in both **3a** and **3b**, hence they show equivalent electronic properties. It is, however, envisaged that **3a** has a strong tendency to aggregate in solutions. For ease of electrochemical and optical characterization as demonstrated in the following, **3b** was therefore prepared by introducing several hexyloxy substituents on the TPA moieties. All of these BDF-TPA triads have been characterized by NMR and Mass spectroscopy. Compound **2** crystallizes as solvate-free colorless needles in the monoclinic space group *P*2_1_/n. In one unit cell, there are two independent molecules situated on a crystallographic center of symmetry. As illustrated in Figure 2, the BDF core is planar, and the dihedral angles formed between the phenyl rings directly linked to BDF core and the BDF unit is 56.4°. There are no exceptional geometrical features, and all bond lengths and angles are within the expected range; they compare well with reported structures of BDF and TPA derivatives. There are no strong intermolecular interactions such as π-π interactions because of the steric effects of the two TPA groups.

### Electrochemical Properties

**FIGURE 3:**
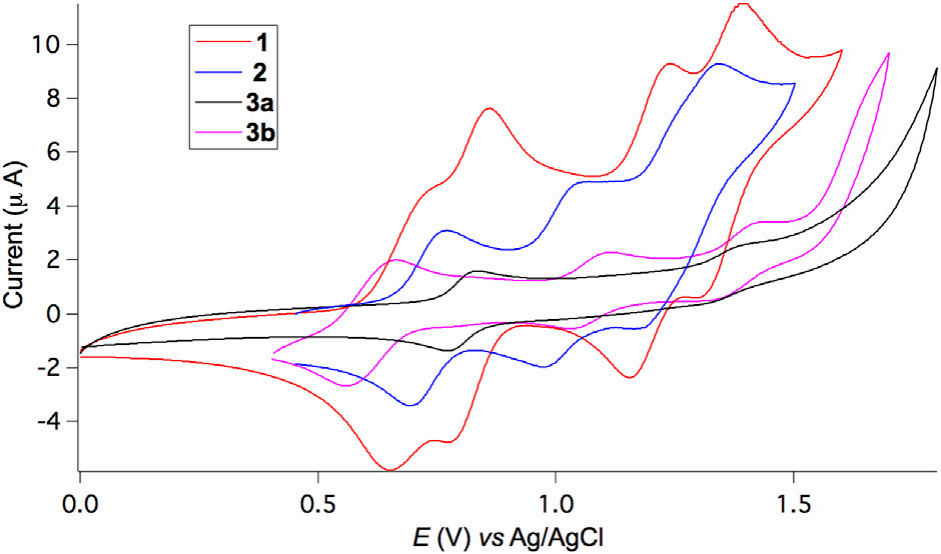
Cyclic voltammograms *versus* Ag/AgCl of **1**, **2,** and **3** in CH_2_Cl_2_ and 0.1 M Bu_4_NPF_6_ supporting electrolyte at a scan rate of 100 mV/s.

The redox properties of triads **1−3** were investigated by cyclic voltammetry (CV) in CH_2_Cl_2_. As depicted in Figure 3, triad **1** undergoes four distinct, reversible single-electron oxidation processes with the corresponding redox potentials of 0.69, 0.82, 1.20, and 1.35 V against an Ag/AgCl reference electrode. For non-conjugated **2**, three reversible single-electron oxidation waves appear at very similar but slightly higher potentials of 0.73, 1.00, and 1.24 V compared to **1**, indicative of the lack of strong electronic communications between BDF and TPA units. In contrast, **3b** shows likewise three reversible oxidation waves at very similar but slightly lower potentials of 0.55, 1.08, and 1.38 V, owing to the presence of electron-donating hexyloxy substituents and extended π-conjugation. Two electrons are likely involved during the first oxidation process. For **3a**, it is impossible to obtain well-resolved redox peaks, presumably due to strong aggregation of the π-extended BDF skeleton without the presence of hexyloxy groups. Both BDF and TPA subunits are electron donating groups with oxidation potentials very close to each other. Consequently, the sequence of oxidation steps for such triads is not obvious.

Furthermore, the conjugated double bonds of the linkers in **1** and **3** lead to comparatively strong electronic interactions between the relevant redox moieties and the description of the oxidation processes as being localised on a given fragment may not be adequate. In this case, charge-transfer processes are expected to be more complex. Spectroelectrochemistry has been used to unravel the sequence of events (*vide infra*).

### Absorption and Photoluminescence Properties in Solution

**FIGURE 4:**
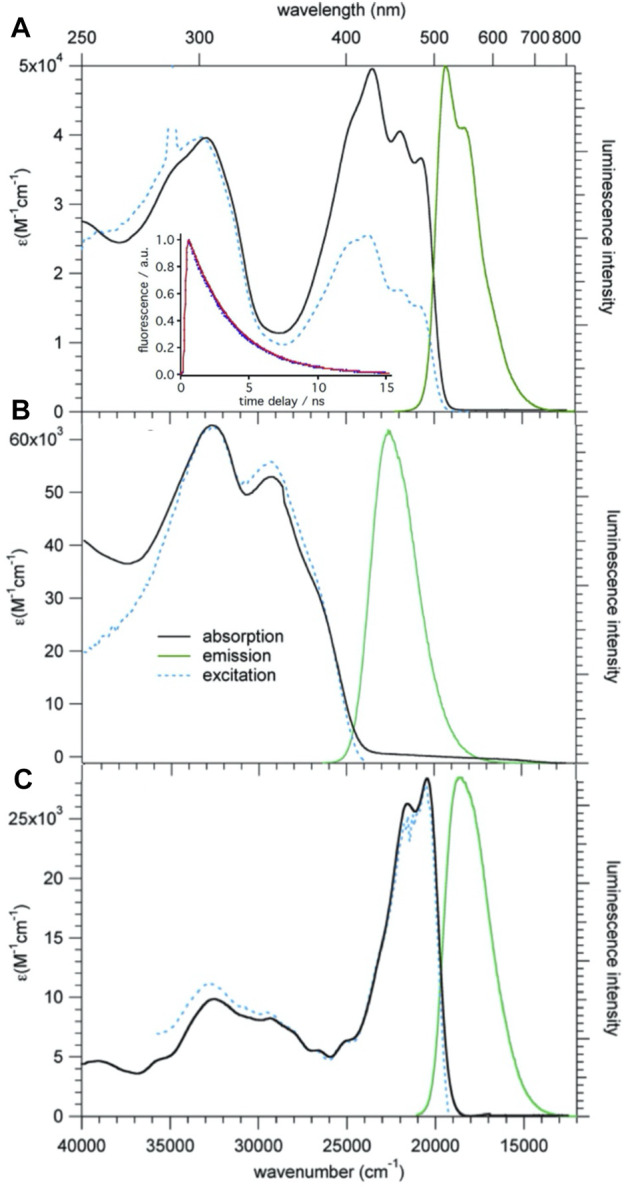
Absorption, emission, and excitation spectra of **1 (A)**, **2 (B)**, and **3b (C)**, *c* = 10^−5^ M in CH_2_Cl_2_ solution at room temperature; for the emission and excitation spectra of **1** and **3b,** λ_ex_ = 430 nm and λ_em_ = 550 nm, respectively, for **2** the corresponding values are 320 and 450 nm, respectively. Inset: luminescence decay of **1**, experimental: blue, single exponential fit: red.

Figure 4A shows UV-vis absorption, photoluminescence (PL) and excitation spectra of **1** in CH_2_Cl_2_ at room temperature. The absorption spectrum consists of a broad structured band with maxima at 20,600, 22,000, and 23,500 cm^−1^, and a broad unstructured band centered at 32,800 cm^−1^. On excitation at 430 nm (23,000 cm^−1^) compound **1** shows an intense, only slightly structured PL band centered at 19,500 cm^−1^ (530 nm). The perfect superposition of the absorption spectrum with the excitation spectrum indicates that the PL is indeed intrinsic to the molecular triad and not due to impurities. The absorption and PL spectra do not have a mirror-image relationship. This is due to the contribution of several electronic transitions to the absorption band. The PL lifetime as measured in deoxygenated solution at room temperature upon pulsed excitation at 395 nm is 3.2(1) ns (see Figure 4A, inset), and the quantum efficiency is 0.77, giving a radiative lifetime of 4.2 ns.

The assignment of the lowest energy absorption bands can be based on TD-DFT calculations performed on **1** using the B3LYP functional and the DZVP2 basis set implemented in the NWChem program package (Aprà et al., 2020). In particular the lowest energy S_0_ → S_1_ transition calculated at 20,700 cm^−1^ (483 nm), has very high oscillator strength and corresponds with 98% to a one-electron transition during which the electron is promoted from the highest occupied MO (HOMO) to the lowest unoccupied MO (LUMO). Both are extended over the whole molecule due to the conjugated double bonds linking the three moieties (Supplementary Figure 1). The lowest energy excitation is thus best identified as π-π* transition with at most a partial charge localisation on the BDF core in the excited state. The calculated Franck-Condon energy of this transition is in very good agreement with the lowest energy band in the experimental spectrum, and the optical HOMO-LUMO gap according to DFT is computed at 2.82 eV = 22,750 cm^−1^. Above the first intense band, several weaker transitions follow, giving rise to the observed structure in the lowest energy band. The next higher energy transitions with large oscillator strength are the S_0_ → S_7_ and S_0_ → S_8_ transitions calculated at around 30,000 cm^−1^ (330 nm), that is, close to the higher energy band in the experimental absorption spectrum. The overall agreement between the experimental and the calculated absorption spectrum of **1** is very good.

Figures 4B,C show the absorption emission and excitation spectra for compounds **2** and **3b**. The lowest energy transition of **3b** is at very similar energies as for **1** and the luminescence quantum efficiency is with 0.75 also quite similar. For **2** the lowest energy absorption band is at substantially higher energy. This is attributed to the fact that without the vinyl linker, the phenyl ring of the TPA linked to BDF is no longer co-planar to the latter, and that therefore the π-system is less extended. This observation is consistent with sizable dihedral angles as shown in its single crystal structure. The corresponding luminescence is likewise blue-shifted and the quantum efficiency is 0.48.

### Spectroelectrochemistry

The CV of **1** inside the spectroelectrochemical cell (Supplementary Figure 2) shows the same four oxidation waves as the high-quality voltammogram. For the Ag wire pseudo-reference electrode used in the spectroelectrochemical cell, the onset of the oxidation occurs at approximately 100 mV lower potential than for the Ag/AgCl reference used for recording the high-quality CV.

**FIGURE 5:**
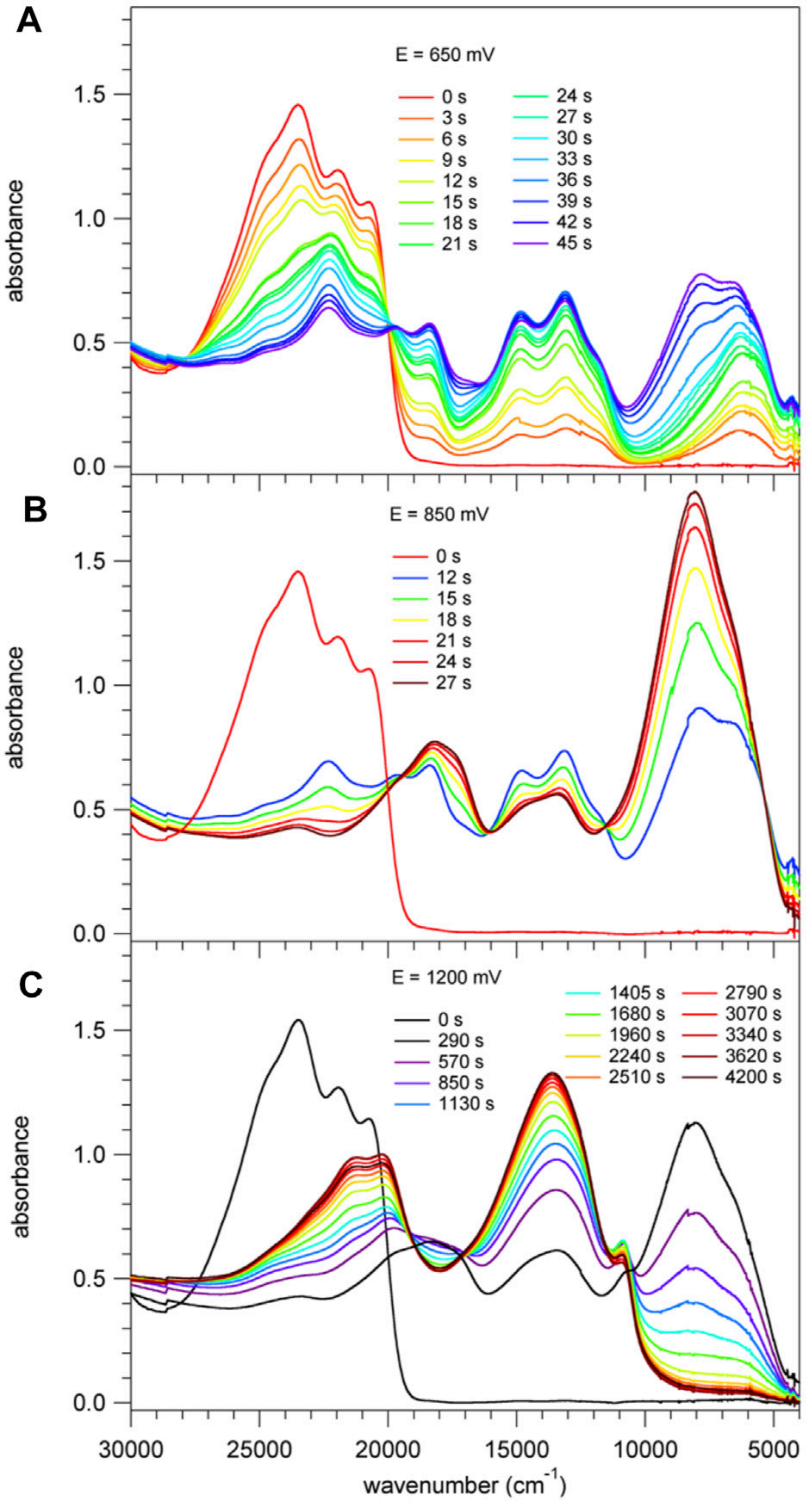
Absorption spectra of **1**, *c* **=** 2 × 10^−4^ M in CH_2_Cl_2_ containing 0.1 M of TBAPF_6_. These spectra were recorded right after applying for a specific time a positive potential of **(A)** 650 mV, **(B)** 850 mV and **(C)** 1200 mV against an Ag wire pseudo reference electrode.

**FIGURE 6:**
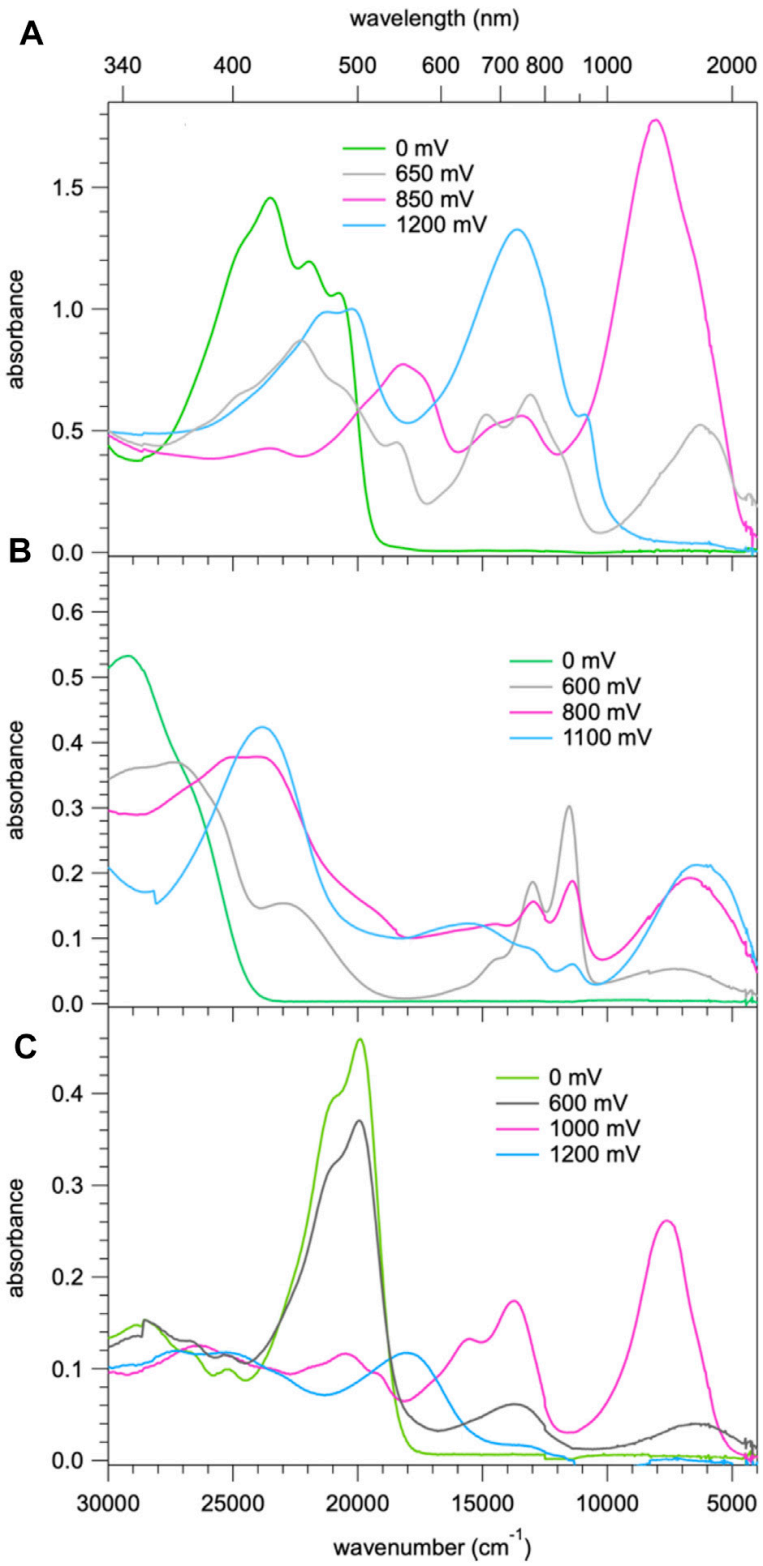
**(A)** Absorption spectra of **1** corresponding to the first three oxidation steps with the most probable spectra of the three species extracted from the spectra of Figure 4; **(B)** absorption spectra of **2** corresponding to the first three oxidation steps with the most probable spectra of the three species; **(C)** absorption spectra of **3b** corresponding to the three species on sequential oxidation.

Figure 5 shows the spectral evolution of **1** with an applied positive potential vs. the silver wire pseudo reference electrode. The spectrum before applying any potential consists of the abovementioned structured band with a maximum 23,250 cm^−1^ (430 nm) and no absorption in the visible range below 20,000 cm^−1^ and in the NIR. At an applied potential of 650 mV (Figure 5A) a broad unstructured band appears rapidly in the NIR with a maximum at around 6,000 cm^−1^ (1650 nm) and a weaker shoulder on the low-energy side, that is at 4,200 cm^−1^ (2,400 nm, Supplementary Figure 3 showing the spectra on a wavelength scale for better visibility of this band). Concomitantly, a second, more structured feature appears in the region of 16,660 – 11,800 cm^−1^ (600 – 850 nm) and the intensity of the original band structure at around 23,000 cm^−1^ nm decreases rapidly. Upon prolonged application of 650 mV a second broad band and more intense band centred at 8,000 cm^−1^ (1250 nm) starts to appear slowly on the high-energy side of the band at 6,000 cm^−1^. On applying a potential of 850 mV (Figure 5B) to a fresh solution, this second band, in turn, increases rapidly at the expense of the band at 6,000 cm^−1^. At the same time the shoulder at 4,200 cm^−1^ disappears again. Finally, application of 1200 mV (Figure 5C) to yet again a fresh solution results in a very rapid appearance of the spectrum obtained at 850 mV followed by a decrease of the intense band at 8,000 cm^−1^ to zero intensity and the appearance of strong bands at 13,000 cm^−1^ (770 nm) and 21,000 cm^−1^ (475 nm). Clearly, the oxidation up to a potential of 1200 mV occurs in three distinct steps. The corresponding most likely spectra of the three species extracted from Figure 5 are shown in Figure 6A and Supplementary Figure 4. The spectrum appearing at a potential of 650 mV corresponds to the one-electron oxidised form **1** ^**+**^. Even though the HOMO is delocalised, the interaction of the oxidised molecule with the weakly polar solvent will quickly result in the localisation of the positive charge on one of the subunits with the inherently lowest oxidation potential, that is, on one of TPA units. In the absorption spectrum following the first oxidation, the intense absorption band at 6,000 cm^−1^ (1650 nm) corresponds therefore to BDF to TPA^+^ charge-transfer transition. The weaker band at 4,200 cm^−1^ (2,400 nm) can be associated with a photo-induced hole transfer from the TPA^+^ to the neutral TPA, that is an intervalence transition (IV-CT) (Lambert et al., 2002). On the second oxidation step, the intense band at 6,000 cm^−1^ loses its intensity at the expense of new even more intense band at 8,000 cm^−1^ (1250 nm). This is due to the oxidation of the second TPA, which results in the shift to higher energy and an increase in intensity of the BDF to TPA^+^ charge-transfer transition. At the same time this also explains the disappearance of the band associated with an intervalence transition. In the third oxidation process, BDF is oxidised to BDF^•+^ and the BDF to TPA^+^ absorption band is fully replaced by the new intense band at 13,000 cm^−1^ (770 nm). The nature of this transition is not entirely clear, it could be a delocalised SOMO to LUMO transition or BDF^+^-related transitions (Shukla et al., 2008; Keller et al., 2011).

For compound **3b**, the evolution of the spectrum on oxidation shows similar three steps as for compound **1**. Figure 6C shows the most probable spectra of the species associated with the three oxidation steps (for the full evolution of the spectrum, see Supplementary Figure 5). As the potential shifts positively, a BDF to TPA^+^ charge-transfer transition around 5500 cm^−1^ initially appears and is subsequently shifted to 7,500 cm^−1^ which then completely disappears, concomitantly with the appearance of a new absorption band at 23,000 cm^−1^. In the last step, the BDF is oxidised to BDF^+^ species. The absence of an IV-CT transition indicates the two TPA units are oxidised simultaneously, which is in good agreement with its CV results.

For compound **2**, the evolution on oxidation is somewhat different. The most likely spectra of intermediate species are given in Figure 6B (for the full evolution of the spectrum, see Supplementary Figure 6). Upon the first oxidation, two well-defined absorption bands at 13,000 cm^−1^ (770 nm) and 12,000 cm^−1^ (830 nm), and one broad band at 7,000 cm^−1^ (1430 nm) appear. The former underpins clearly the signature of the BDF^+^ radical species (Shukla et al., 2008; Keller et al., 2011) whereas the latter is very probably due to a TPA to BDF^+^ charge-transfer transition. As the oxidation proceeds, an IV-CT band around 6,500 cm^−1^ (1540 nm) is observed, indicative of the participation of one TPA unit in the process. During the third oxidation, the intrinsic absorption bands of the radical cation BDF^•+^ significantly decrease and a new intense band at 24,000 cm^−1^ (420 nm) appears, which might be attributed to the formation of BDF^2+^ dication species.

### Photoluminescence in Solid Films and OLED Performance

**FIGURE 7:**
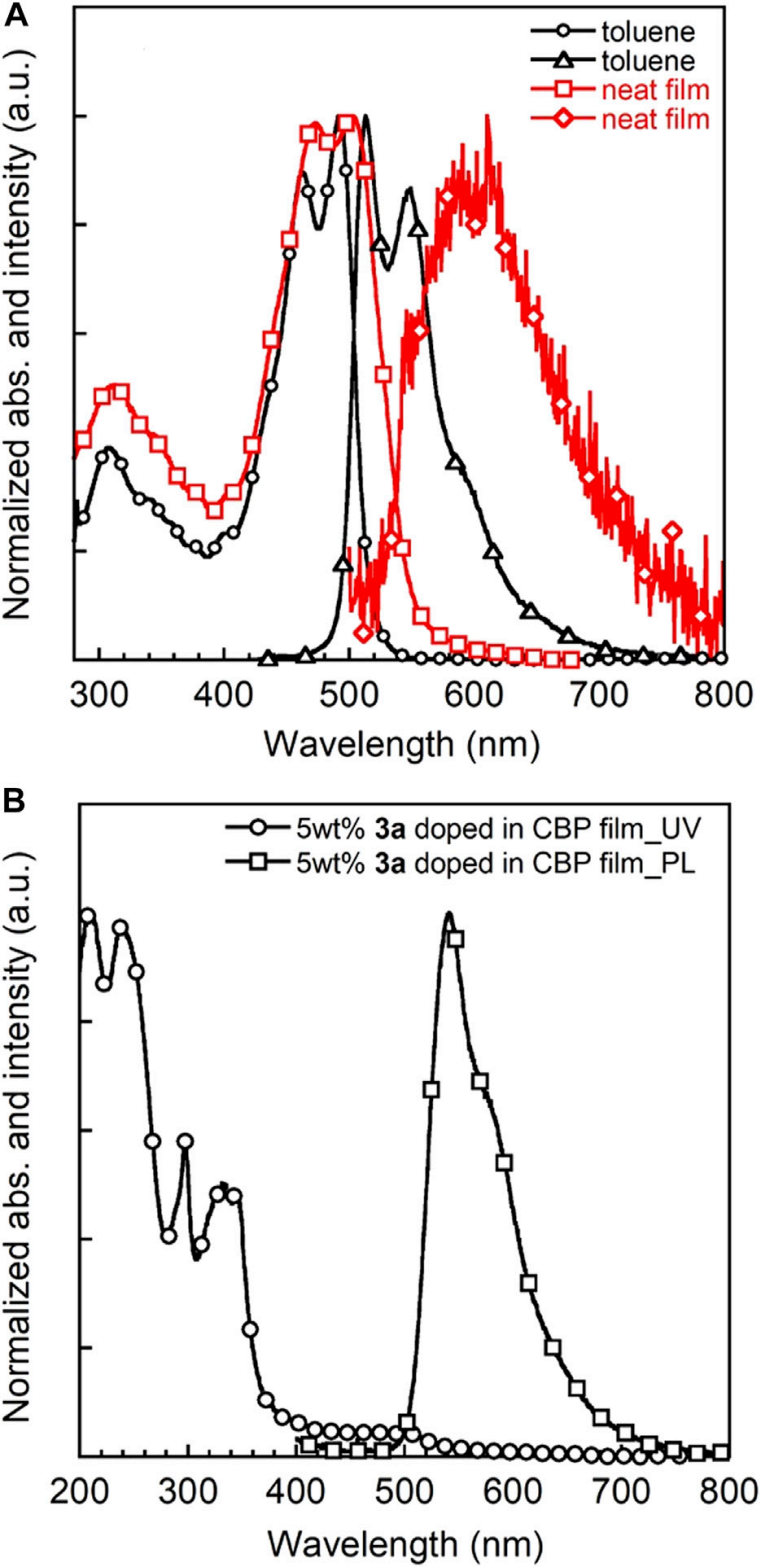
UV-vis and PL spectra of **3a** in toluene (*c* = 1 × 10^−5^ M) and neat film **(A)** and 5 wt% **3a**-doped CBP film **(B)**.

The UV-vis absorption and PL spectra of **3a** in toluene solution, a neat film and a 5 wt% **3a** doped CBP film are depicted in Figure 7. Triad **3a** shows intense optical absorptions over the UV-vis spectral part similar to **3b**, with absorption onset energies at about 520 nm (19,200 cm^−1^) and 580 nm (17,200 cm^−1^) for solution and film, respectively. The λ_abs,max_ of the **3a** film appears at a longer wavelength than that in solution, most likely due to the aggregation in the solid state. Moreover, **3a** exhibits a strong orange emission with a PL quantum efficiency (PLQE) of 70% in toluene, comparable to **3b** in CH_2_Cl_2_ and other BDF derivatives (Keller et al., 2011; Li et al., 2013a; Yi et al., 2010). A very small stokes shift of 22 nm (880 cm^−1^) is observed probably because of the rigid molecular structure. The optical band gap is determined to be 2.3 eV from the intersection of the absorption and PL spectra in a neat film. Taken together with CV data, the LUMO energy level is estimated to be −2.7 eV. The ionisation potential (IP) value of a **3a** neat film was also determined by photoelectron yield spectrometry to be around 5.2 eV (Supplementary Figure 8 and Supplementary Table 3), which matches well with the HOMO energy level (5.0 eV) estimated from CV measurements. The same holds true for IP values and HOMO energy levels of triads **1** and **2**. Although the 5 wt% **3a** doped CBP film shows a similar absorption spectrum as the host molecule CBP in the film (Gong et al., 2012), no CBP-based emission at 400 nm but a strong PL from **3a** chromophore was observed, indicating efficient energy transfer from host CBP to **3a**. Additionally, PLQEs of **3a**-doped CBP films were recorded to be around 60% for 1 wt% and 5 wt%, which are significantly higher than those of 5 wt% **1** and **2** doped CBP films (32 and 47%, respectively, Supplementary Table 3). Notably, PLQEs are greatly decreased with increase of **3a** concentration (Supplementary Figure 7 and Supplementary Table 2) indicative of the strong aggregation of the π-extended BDF skeleton.

**FIGURE 8:**
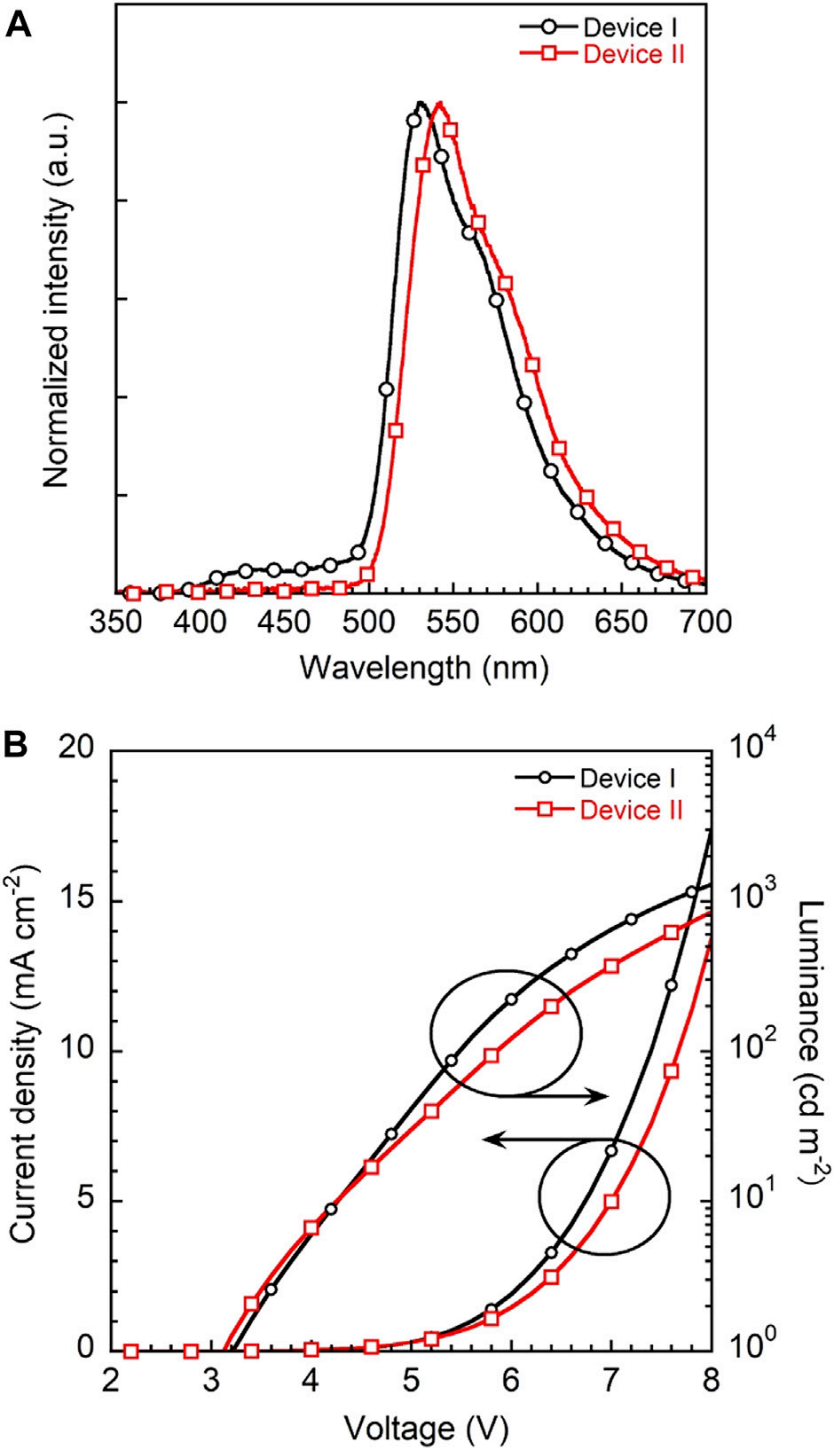
**(A)** EL spectra of devices I and II with 1 and 5 wt% **3a**-doped CBP as an emissive layer, respectively and **(B)** current density-voltage-luminance (J-V-L) plots of two devices.

To evaluate the device performance, two types of **3a** based OLEDs were fabricated, (I) 1 wt% **3a**-doped CBP and (II) 5wt% **3a**-doped CBP as an emission layer (EML), respectively. The configuration of the OLED devices is designed as ITO (130 nm)/PEDOT:PSS (40 nm)/EML (30 nm)/bis-4,6-(3,5-di-4-pyridyl-phenyl)-2-methylpyrimidine (B4PYMPM) (50 nm)/Liq (3 nm)/Al (100 nm) (Figure 1). The current density–voltage–luminance (I–V–L) and electroluminescence (EL) spectra of two devices are illustrated in Figure 8, device characteristics (Supplementary Figure 9) are summarised in Table 1 and these data were calculated from Lambertian assumption.

**TABLE 1:** Characteristics of OLED devices I (1 wt%) and II (5 wt%).

Device	At 1 cd/m^2^		At 100 cd/m^2^			At 1000 cd/m^2^			CIE (x,y)	
	D.V.	E.Q.E	D.V.	P.E.	E.Q.E	D.V.	P.E.	E.Q.E				
	(V)	(%)	(V)	(Im/W)	(%)	(V)	(Im/W)	(%)				
I	3.2	6.2	5.5	7.1	3.5	7.6	3.4	2.3	0.37	0.58
II	3.1	4.9	5.8	4.6	2.4	8.2	2.2	1.6	0.41	0.57

The EL spectra of devices I and II with a λ_max_ at 530 nm are quite similar to the corresponding PL spectra of the emission layers on their own (Supplementary Figure 10). Both devices show a yellowish-green emission with CIE_x,y_ coordinates of (0.37, 0.58) and (0.41, 0.57) and low turn-on voltages of 3.2 and 3.1 V, respectively. However, device I features a higher maximum brightness of 1290 cd m^−2^ at 8 V than device II with 844 cd m^−2^.

Notably, the EQEs in both devices (Supplementary Figure 9) are higher than the theoretical limit. In particular, device I exhibits an EQE of 6.2%, which is 35% higher than the theoretical maximum of about 4.6% as calculated according to the equation EQE(η_ext_) = γ×η_r_×η_PL_×η_out_, *γ*: charge balance (∼100%)_,_ η_r_: exciton formation ratio (25% for fluorescence, 100% for phosphorescence), η_PL_: PLQE (61%) and η_out_: outcoupling efficiency (∼30%). Kim *et al.* demonstrated that energy transfer from an exciplex formed between the host material CBP and the electron transporting layer bis-4,6-(3,5-di-3-pyridylphenyl)-2-methylpyrimidine (B3PYMPM) to the dopants enhanced the efficiency significantly (Park et al., 2011). Recently, they could make use of energy transfer from the host exciplex to the phosphorescent dopant, finally achieving high performance OLEDs (Park et al., 2013). The same holds for our present case. The EL spectrum of device I shows two emission peaks at 530 nm from **3a** and a weaker one at 430 nm from a CBP/B4PyMPM exciplex (Figure 8). In contrast, the corresponding EL spectrum of device II displays no emission around 430 nm, probably due to complete energy transfer from the exciplex to the EML with a higher concentration of dopant.

**FIGURE 9:**
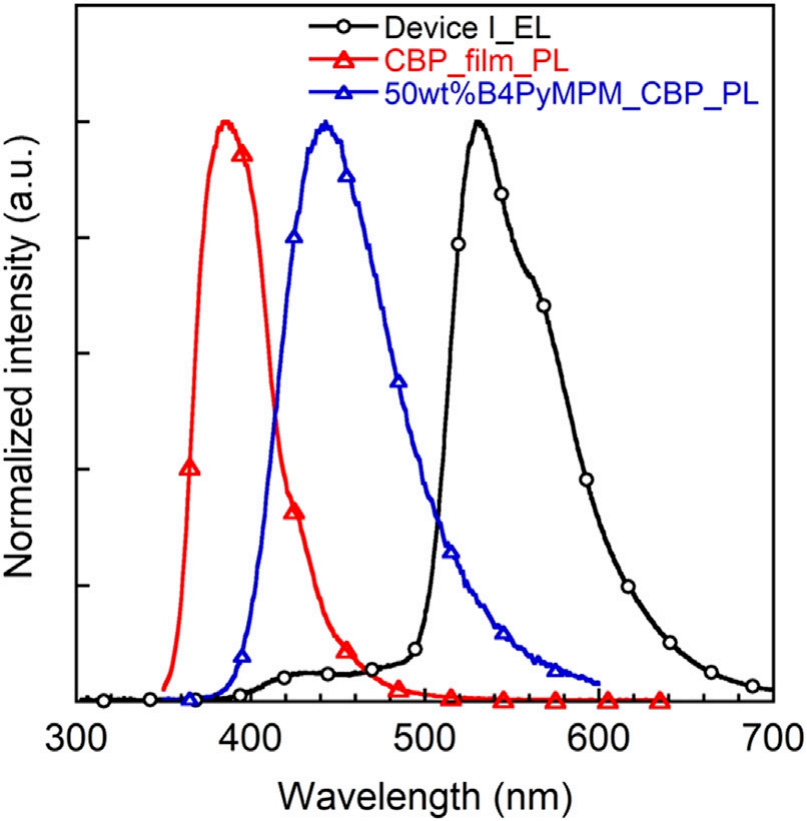
EL spectrum of device I and PL of CBP neat film and CBP/B4PyMPM blend.

To verify this speculative emission peaking around 430 nm from the exciplex, the PL spectrum of a co-evaporated CBP/B4PyMPM blend with a weight ratio of 1:1 was recorded. As expected, an exciplex emission around 440 nm (Figure 9), which is red-shifted compared to either CBP or B4PYMPM emission band, matches well with our observations in the EL spectrum of device I. The energy level of the exciplexes is 2.8 eV, which is similar to the difference between the HOMO level of the CBP and the LUMO level of the B4PYMPM, which underlines that the CBP and B4PYMPM molecules form an exciplex upon excitation. Recently, Zhao and co-workers reported that oligofluorene-based emitters in CBP host films fabricated by spin-coating method exhibit moderate horizontal orientation (orientation parameter, *S* ∼ −0.3) for those exhibiting a large molecular shape anisotropy (Zhao et al., 2016). Therefore, another possible mechanism for this enhancement in EQE is the contribution from the horizontal molecular orientation because **3a** has a relatively large molecular shape anisotropy similar to oligofluorene-based emitters, and we also used CBP as a host material in solution-processed OLEDs. However, at this stage, we are not able to quantify the contributions from the energy transfer from exciplex and/or the horizontal molecular orientation of the emitter molecule.

## Conclusion

We have developed facile synthetic routes to triphenylamine-substituted benzodifuran triads **1–3**. All of them have been fully characterized, and their electronic absorption and PL properties as well as redox behaviour have been investigated in detail. Compared to non-planar **2**, π-extended conjugation *via* vinyl linkers leads to the coplanarity between the relevant redox TPA and BDF moieties in **1** and **3**, with comparatively strong electronic interactions. Due to intriguing electronic properties and unique structural features, the OLED device with 1 wt% **3a**-doped CBP as an emissive layer achieves a high external quantum efficiency (EQE) of 6.2%. Given such promising results, **3a** can serve as an attractive dopant emitter in OLEDs, and our present study should assist in future research in the development of efficient fluorescent chromophores by emphasizing the full use of energy transfer from an exciplex to an emissive layer.

## Data Availability

The original contributions presented in the study are publicly available. This data can be found here: CCDC 2062823 contains the supplementary crystallographic data for 2. These data can be obtained free of charge *via* www.ccdc.cam.ac.uk/data/cif, or by emailing data_request@ccdc.cam.ac.uk, or by contacting The Cambridge Crystallographic Data Centre, 12 Union Road, Cambridge CB2 1EZ, United Kingdom; fax: +44 1223 336033.
