# Supplementary material for: Bis(Triphenylamine)Benzodifuran Chromophores: Synthesis, Electronic Properties and Application in Organic Light-Emitting Diodes
Source: Front Chem. 2021 Jul 21;9:721272. doi: 10.3389/fchem.2021.721272 (PMC8333860; doi:10.3389/fchem.2021.721272)
Supplement: Supplementary file 1 [file DataSheet1.PDF]

frontiers

Supplementary Material

1.1 Supplementary Figures

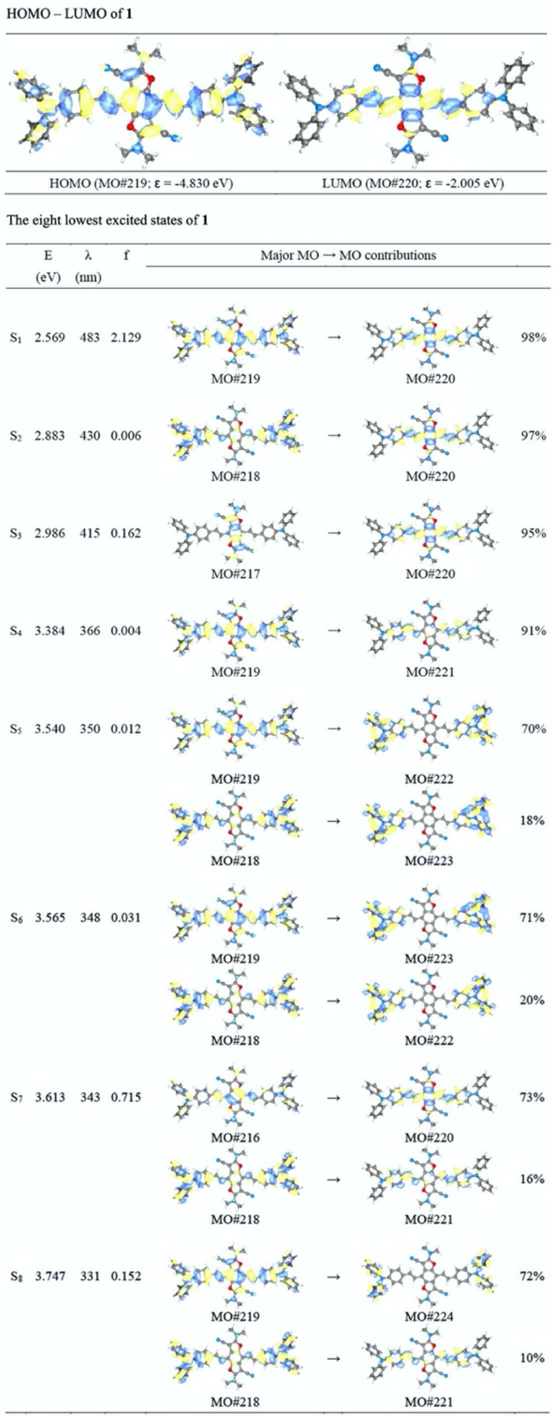

Supplementary Figure 1. HOMO – LUMO and the first eight excited states of **1** calculated with DFT.

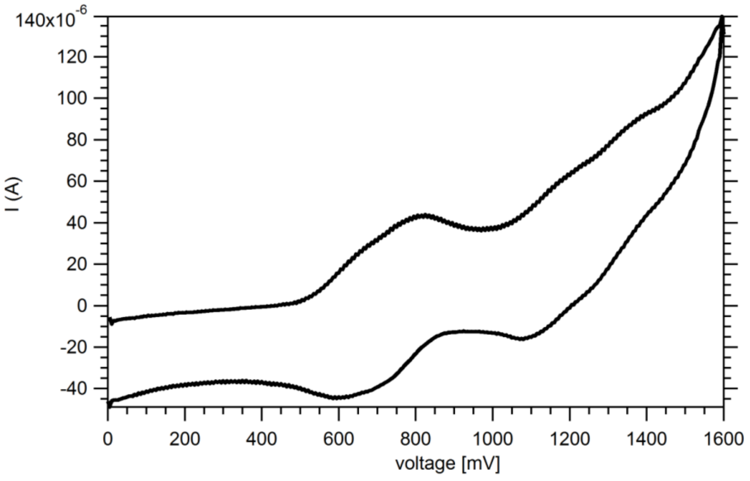

Supplementary Figure 2. Cyclic voltammogram of **1** (2×10^{-4} M) recorded inside the spectro-electrochemical cell. Scan rate = 0.1 V/s, Ag wire pseudo reference.

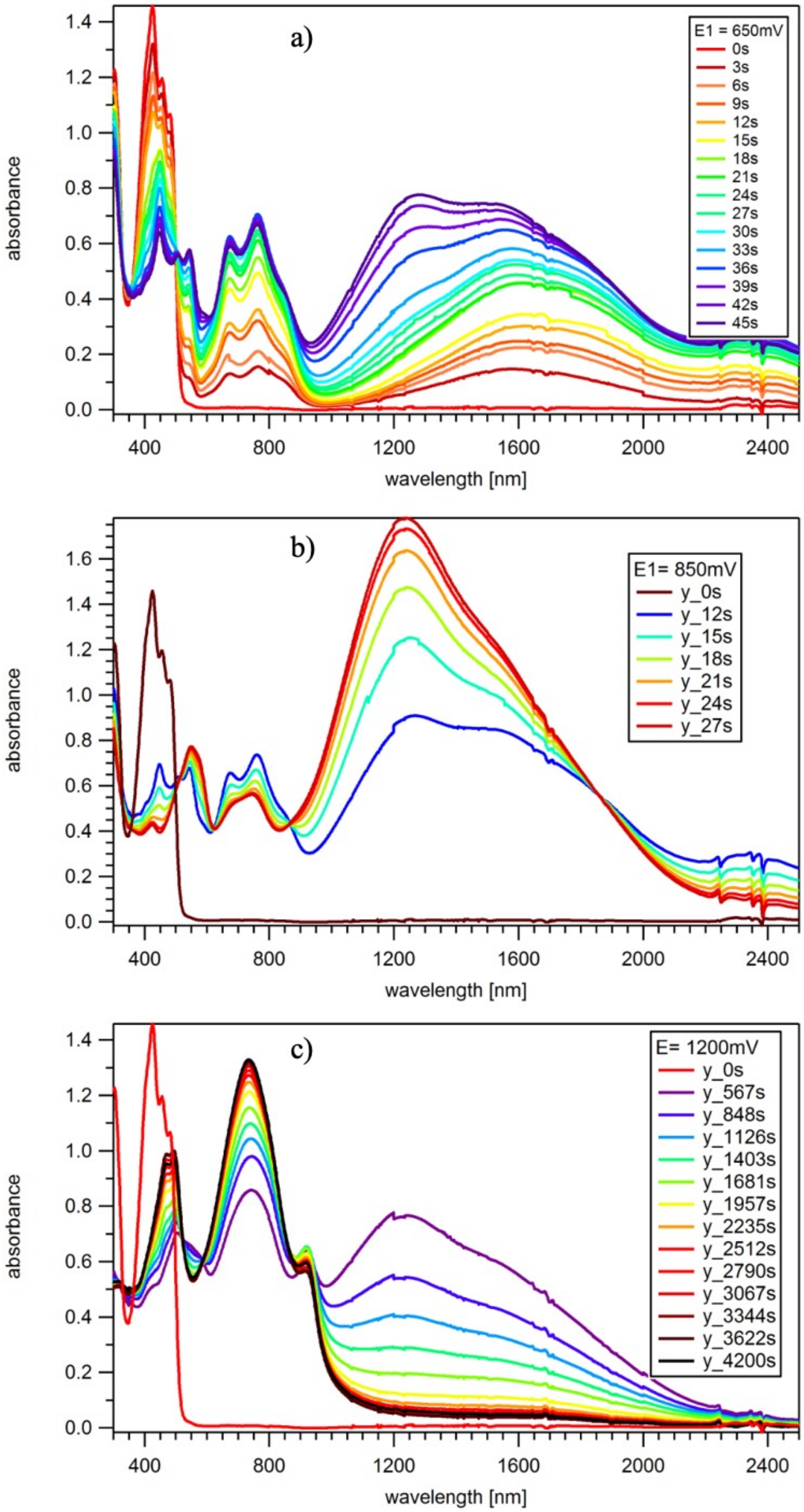

Supplementary Figure 3. Absorption spectral change of **1** (2×10^{-4} M) in CH_2Cl_2 containing 0.1 M of $TBAPF_6$. These spectra were recorded right after applying for a specific time a positive potential of (top) 650mV, (middle) 850 mV and (bottom) 1200 mV.

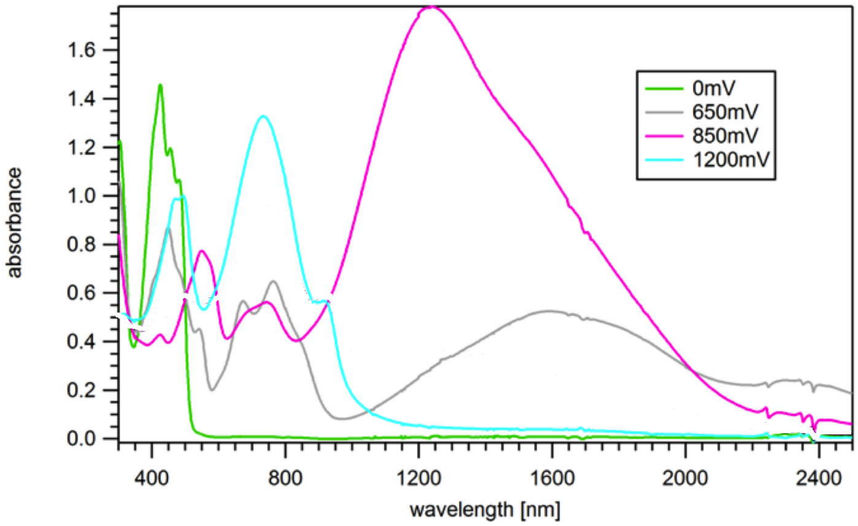

Supplementary Figure 4. Absorption spectra of **1** corresponding to the cleanest oxidation steps with the spectra of the three species.

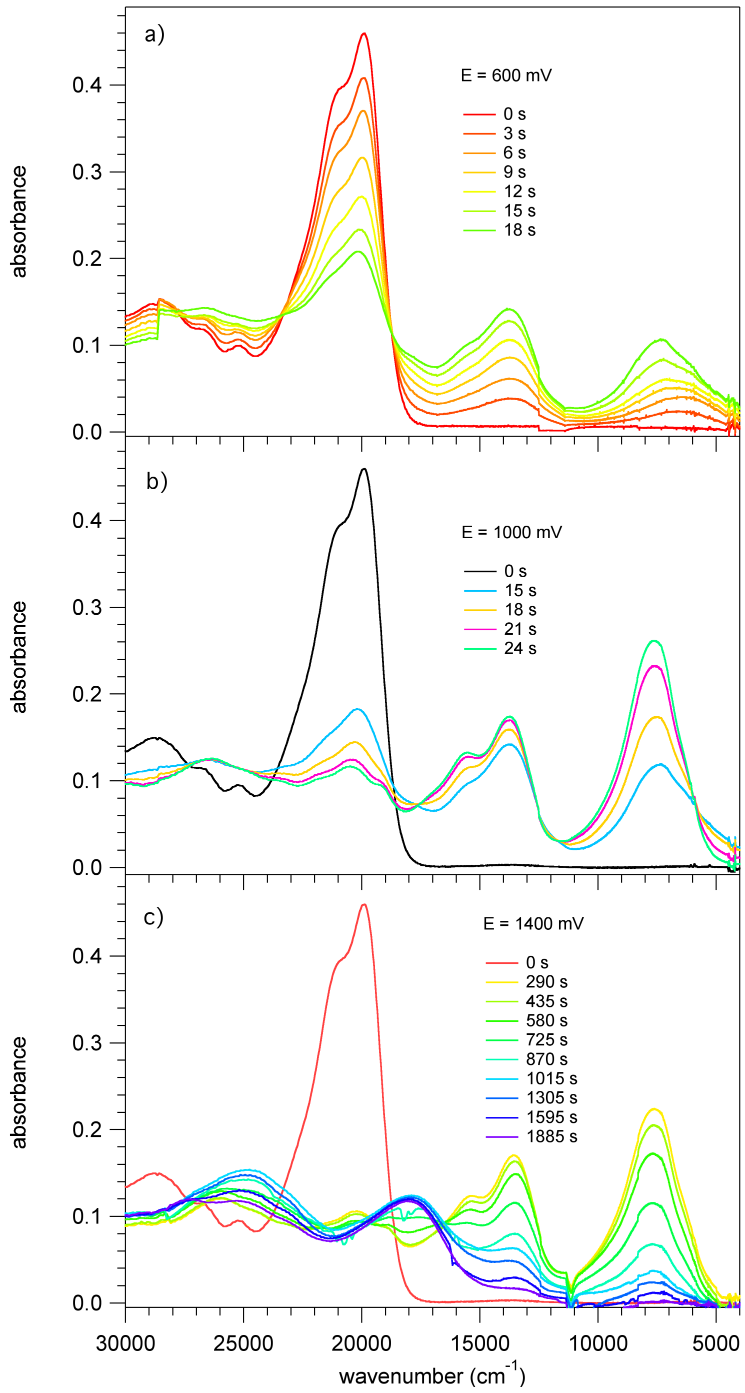

Supplementary Figure 5. Absorption spectra of **3b**, $c = 8\times10^{-5}$ M in CH_2Cl_2 containing 0.1 M of $TBAPF_6$. These spectra were recorded right after applying for a specific time a positive potential of (a) 600 mV, (b) 1000 mV and (c) 1400 mV against an Ag wire pseudo reference electrode.

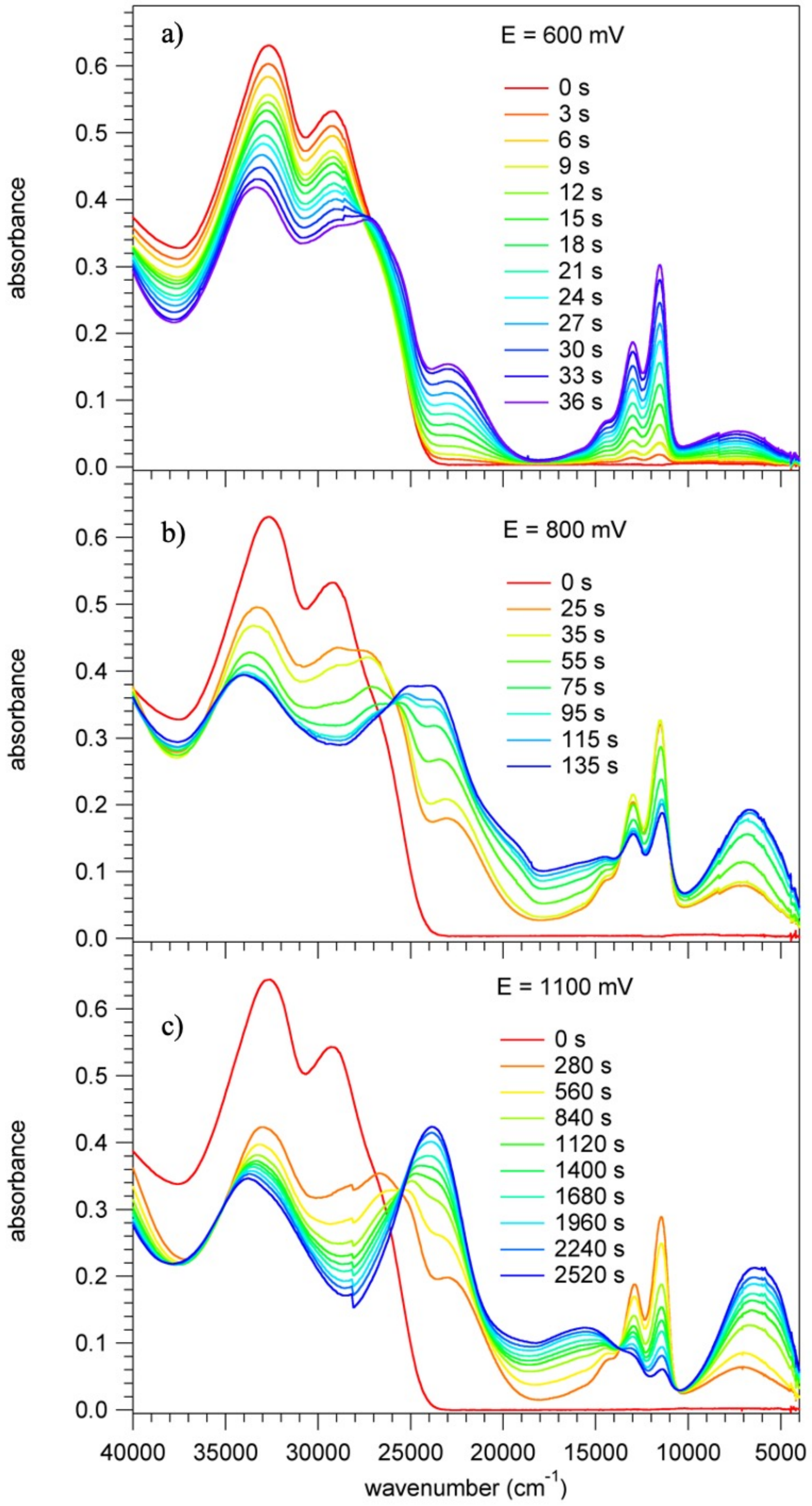

Supplementary Figure 6. Absorption spectra of **2**, $c = 1\times10^{-4}$ M in CH_2Cl_2 containing 0.1 M of $TBAPF_6$. These spectra were recorded right after applying for a specific time a positive potential of (a) 600 mV, (b) 800 mV and (c) 1100 mV against an Ag wire pseudo reference electrode.

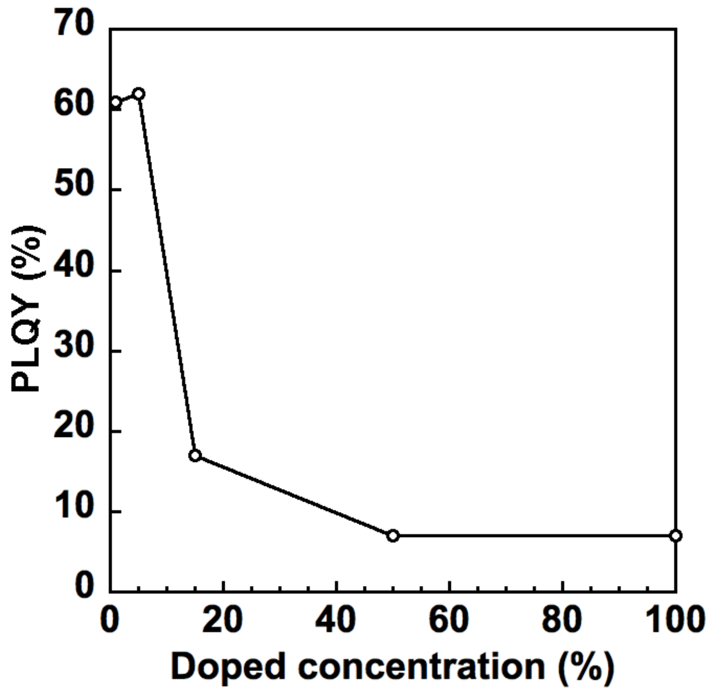

Supplementary Figure 7. PLQE of **3a**-doped CBP film at λ_{ex} of 330 nm.

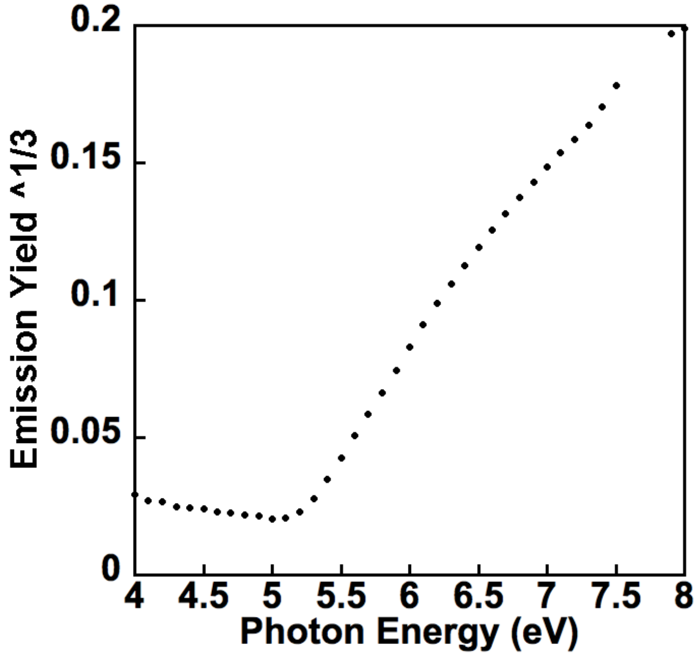

Supplementary Figure 8. Photoemission yield spectrum of **3a**-neat film.

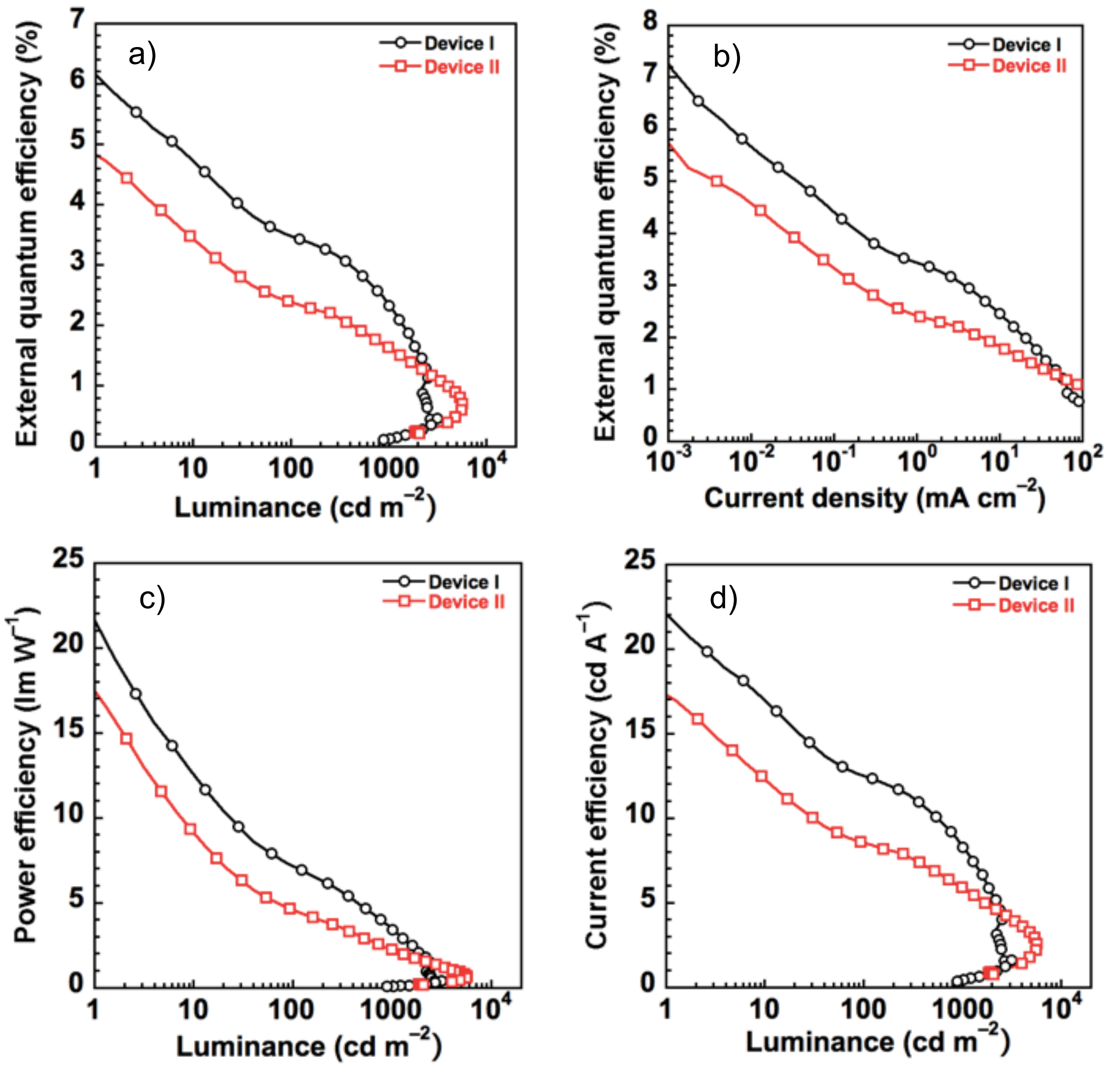

Supplementary Figure 9. EQE-L characteristics (a) and EQE-current density (EQE-J) characteristics (b), power efficiency-luminance (PE-L) (c), and current efficiency-luminance (CE-L) characteristics (d) of device I and II.

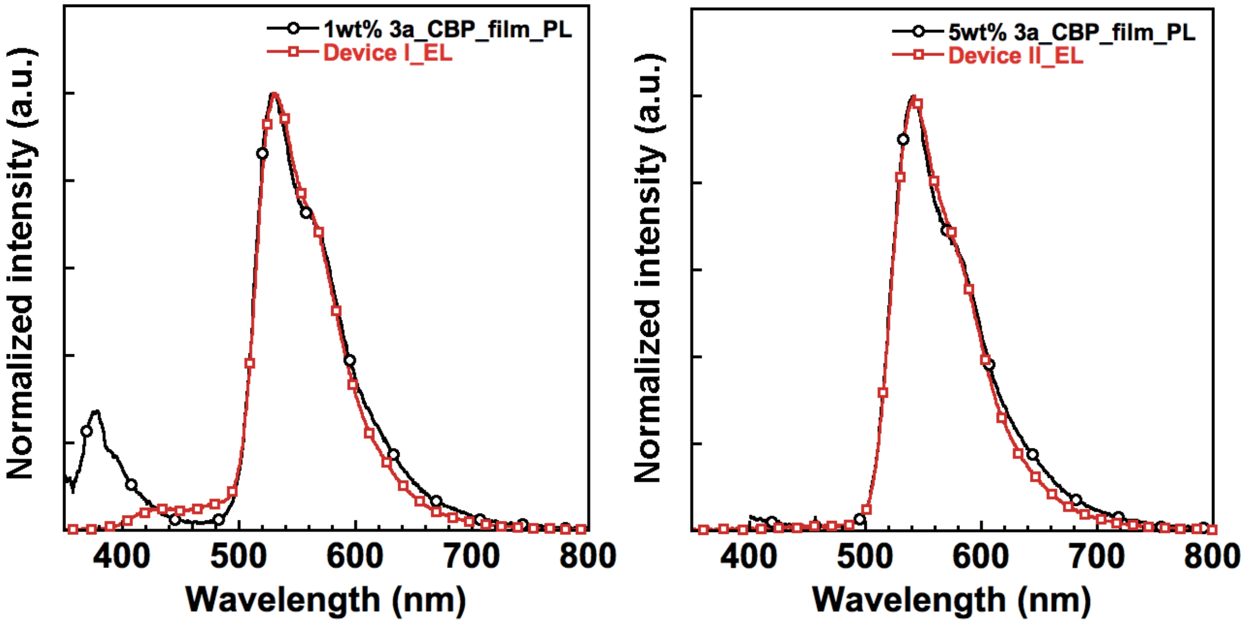

Supplementary Figure 10. EL and PL spectra of device I and II.

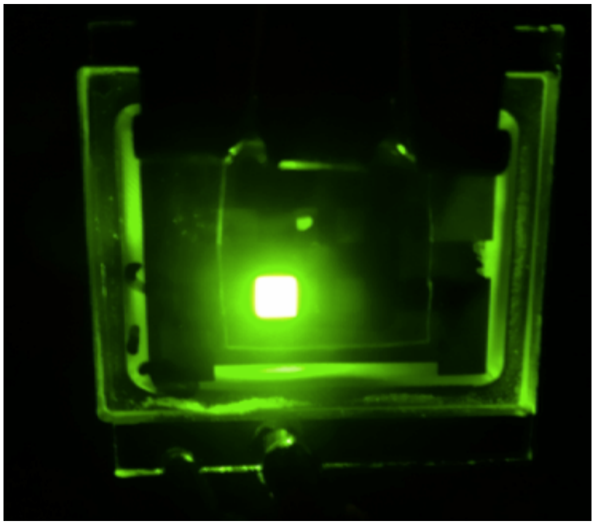

Supplementary Figure 11. Photograph of the emission from a **3a**-based device.

1.2 Supplementary Tables

Table S1 Crystal data and structure refinement for **2**

Empirical formula	C_{72} H_{80} N_6 O_2
Formula weight	1061.42
Temperature	173(2) K
Wavelength	0.71073 Å
Crystal system	Monoclinic
Space group	P 2_1/n
Unit cell dimensions	a = 20.3521(4) Å
	b = 9.8004(2) Å
	c = 30.4970(7) Å
Volume	5971.4(2) Å^3
Z	4
Density (calculated)	1.181 Mg/m^3
Absorption coefficient	0.071 mm^{-1}
F(000)	2280
Crystal size	0.40 x 0.04 x 0.03 mm^3
Theta range for data collection	2.1 to 25.35°.
Index ranges	-24<=h<=24, -11<=k<=11, -36<=l<=36
Reflections collected	62021

Independent reflections	10937 [$R_{(int)}$ = 0.1188]
Completeness to θ = 25.35°	100 %
Absorption correction	Semi-empirical from equivalents
Max. and min. transmission	1 and 0.70502
Refinement method	Full-matrix least-squares on F^2
Data / restraints / parameters	10937 / 0 / 721
Goodness-of-fit on F^2	1.028
Final R indices [$I > 2\sigma(I)$]	R_1 = 0.0852, wR_2 = 0.1859
R indices (all data)	R_1 = 0.1617, wR_2 = 0.2262
Largest diff. peak and hole	0.572 and -0.361 e Å^{-3}

Table S2 PLQE of 1–100 wt% **3a**-doped CBP film at λ_{ex} of 330 nm

Doping concentration (%)	PLQE (%)
1	61
5	62
15	18
50	7
100	7

Table S3 A summary of optical properties of **1**-**3**.

Compound	$\lambda_{abs,max}$ (nm)	$\lambda_{em,max}$ (nm)	PLQE (%)	IP(eV)	EA(eV)	E_g(eV)
**1**-neat film	310, 433	600	7	5.3	3.0	2.3
5 wt% **1**-doped CBP film	333	553	32	–	–	–
**2**-neat film	313	440	27	5.5	2.6	2.9
5 wt% **2**-doped CBP film	332	435	47	–	–	–
**3a**-neat film	472, 501	588, 612	7	5.2	3.2	2.0
5 wt% **3a**-doped CBP film	333, 345	542	62	–	–	–
